# Role of Neuronal Apoptosis in Volumetric Change of Hippocampus in Diabetes Mellitus Type 1: A Predictive Model

**DOI:** 10.5402/2013/958461

**Published:** 2013-07-21

**Authors:** Khadije Foghi, Shahriar Ahmadpour

**Affiliations:** Anatomy Department, Medicine School, North Khorasan University of Medical Sciences, Bojnurd, Iran

## Abstract

*Background*. Neuronal apoptosis is the major cause of diabetes central neuropathy, but its role in volumetric changes of hippocampus has not been clarified. The aims of this study were to assess the role of apoptosis in volumetric changes of dentate gyrus (DG) and CA3 region of hippocampus and to determine a reference point in which these neuropathological changes reach a meaningful level. *Methods and Materials*. Diabetes was induced in male Wistar rats (*N* = 10) by streptozotocin (60 mg/kg). Six weeks after diabetes, verification animals were divided into four groups as follows: diabetic treated with insulin (3–5 U), diabetic treated with vitamin C (80 mg/kg), and diabetic and control groups. At the end of 8 weeks, numerical density of apoptotic neurons and volume of dentate gyrus and CA3 were calculated by stereological methods. *Results*. The number of apoptotic neurons in DG and CA3 in diabetic group showed significant level of difference in comparison with the control (*P* < 0.001). The volume of DG and CA3 in diabetic and vitamin C showed significant level of difference compared with control (*P* < 0.001). *Conclusion*. Our results suggest that DG and CA3 volume reduction begins and progresses independently of neuronal loss.

## 1. Introduction

Diabetes mellitus type 1 (DM1) is an endocrine disorder which is characterized by lack of insulin and hyperglycemia [1]. For a long period, it was believed that the central nervous system (CNS) as an insulin independent organ is spared from diabetic complications; however, in recent decades, studies have provided evidence that indicates the deleterious effects of DM1 on structure and functions of the brain [2–4]. Although the mechanisms through which hyperglycemia might mediate these effects are not completely understood, it seems that hyperglycemia increases oxidative stress and free radicals generation. Increased free radicals damage cellular membrane (lipid per oxidation) and initiates neuronal death signaling pathways [5, 6]. Neuronal apoptosis has been known as the main leading cause of diabetes central neuropathy. It is the common hallmark of diabetes mellitus type 1 (DM1) and neurodegenerative disorders like Alzheimer [5–7]. One of the most sensitive regions of brain to oxidative stress is the hippocampus [8]. It is a complex part of the limbic system which plays a pivotal role in cognitive functions like navigation, memory, and learning in animals and human as well. It is divided into dentate gyrus (DG) and CA_1–3_. Among the hippocampus subfields, DG and CA3 build an essential part of memory and learning circuits [9, 10]. The adverse effects of DM1 on hippocampus have been studied extensively. Experimental studies showed that uncontrolled hyperglycemia increases oxidative stress markers in hippocampus and subsequently accelerates neuronal death in dentate gyrus (DG) and CA3 [8, 11–13]. Additionally, suppressed cell proliferation in DG and morphological changes like simplification/retraction of dendrites of CA3 pyramidal neurons have been reported in experimental model of DM1 [9, 12]. In vivo neuroimaging study (Voxel-based morphometry, VBM) could also demonstrate significant volume reduction of grey matter density of hippocampus in diabetic cases [14]. Although neuronal apoptosis as the main cause of diabetic central neuropathy has been reported in the hippocampus, a great challenge in evaluation of diabetic cases that has remained to be answered is the contribution of neuronal loss in volume reduction of brain particularly in the hippocampus. In other words, most of the experimental studies have dealt with each area of the hippocampus separately, and functional/anatomical connectivity of adjacent regions has been ignored. DG and CA3 are connected directly together and can be considered an anatomical module. Based on the anatomical module concept, two strongly connected regions should respond to an insult contemporarily. So calculated response index of DG/CA3 can be used instead of considering each area separately. The aims of this study were (1) to assess the role of neuronal apoptosis in volumetric change and (2) the efficiency of response index (RI DG/CA3) in interpretation and predicting of DM1-related pathologies of the hippocampus.

## 2. Method and Materials

Male Wistar rats (8 weeks old) were randomly divided into 4 groups (*N* = 10). All rats were maintained in animal house and allowed free access to drinking water and standard rodent diet. In 3 groups, diabetes mellitus was induced by single intraperitoneal (IP) injection of streptozotocin (Sigma) at a dose of 60 mg/kg. The control group only received saline. Animals were considered diabetic with fasting blood glucose level above 250 mg/dL four days after diabetes induction [12, 13]. Six weeks after diabetes approval, diabetic animals were treated as follows: group (1) insulin treated (3–5 U) (NPH, Exir, Iran), group (2) antioxidant treated (vitamin C) (Darou Pahksh, Iran) (80 mg/kg, IP), and group (3) diabetic. The treatments were conducted for 2 weeks. At the end of 8 weeks, animals were anesthetized by chloroform and subsequently 5 mL blood was collected from the heart of each animal in order to measure the plasma Cu level as an oxidative stress marker, and then animals were perfused transcardially with 100 mL of saline followed by 200 mL of fixative containing 2% glutaraldehyde and 2% paraformaldehyde in 0.1 phosphate buffer (pH: 7.4). The adrenal glands of animals were removed and weighed. The brains were removed and postfixed in formalin 10% for two weeks. Serial coronal sections (thickness = 5 and 10 microns) were obtained through the entire rostrocaudal extent of paraffin embedded hippocampi in the left and right hemispheres by a microtome (1212 Ernst Leitz Gmbh Wetzlar, Germany).

### 2.1. Volume Estimation of the Dentate Gyrus and CA3

Serial coronal sections (a thickness of 10 microns) were cut through the hippocampus. The total number and order were noted. The volumes of the hippocampi in 4 groups were estimated using Cavalieri's principle [15, 16]. Systematic random sampling selected sections were processed and stained by Cresyl Violet 1% [17]. The volumes of dentate gyrus and CA3 were estimated by Cavalieri's method as follows:

(1) $$\begin{array}{c} V=T\cdot \frac{a}{p}\cdot \Sigma p, \end{array}$$

where *T* is the thickness, *a*/*p* is the area of each point, and Σ*p* is the total number of hit points.

### 2.2. Assay for Apoptosis

TUNEL assay was done by In Situ Cell Death Detection Kit, POD (Rouch), according to the recommended manual (Rouch). Paraffin sections of hippocampus (5 *μ*m) were treated for apoptosis detection as briefly as follows: deparaffinization, absolute ethanol, graded series of 70–95% 3 minutes, washing in PBS 5 minutes, proteinase K 15 minutes, washing in distilled water ×4 (each 2 minutes), hydrogen peroxide 3%, PBS ×2, reaction mixture at room temperature (60 min), PBS ×3, Convertor-POD 30 minutes in 37°C, PBS ×3, adding DAB 5 min, PBS ×3, counterstaining with Toluidine Blue, washing in running water ×3, graded series Alcohol (70–100), xylene, and mounting.

### 2.3. Estimation of Apoptotic Neurons Density by Physical Dissector

The density of apoptotic neurons in the *granular layer* and* pyramidal layers* was estimated by physical dissector principle. We used an Olympus microscope (BX 51, Japan), equipped with a software program, digital 5 analysis. After registrations of paired samples with 5 *μ*m intervals, counting frame was superimposed on the captured pictures, and counting was performed according to the dissector principle [17]. Apoptotic neurons density in unit volume was estimated by the following formula:

(2) $$\begin{array}{c} NV=\frac{1}{ah}\cdot \frac{\Sigma Q}{\Sigma P}, \end{array}$$

where *N* is the apoptotic neuronal density, *a* is the area of applied frame, is linear magnification, Σ*Q* is the number of apoptotic neurons, and Σ*P* is the number of counted frames.

### 2.4. Plasma Cu Level Determination by Atomic Absorption

The collected blood samples were transferred to the laboratory and centrifuged at 3000 rpm × 10 minute. The plasma in labeled polyethylene free tubes was stored at −20°C. Free plasma Cu levels were measured by spectrophotometer CAT3000.

### 2.5. Response Index (RI)

Based on the anatomical connectivity between the dentate gyrus and CA3 region, we tried to develop a response index between the parameters of volume and neuronal death of the two regions of interest. The ratios of mean volumes of DG to CA3 (*V*_DG_/*V*_CA3_) and numerical density of apoptotic neurons (ND_DG_/ND_CA3_) were calculated (Table 1).

### 2.6. Statistics

All data are expressed as mean ± SD. Comparisons of data between groups were made using one-way ANOVA and Tukey's post hoc test. *P* value less than 0.05 was considered as significant. Correlation between the values was estimated by Pearson's correlation.

## 3. Results

### 3.1. Blood Glucose (BG)

**Figure 1:**
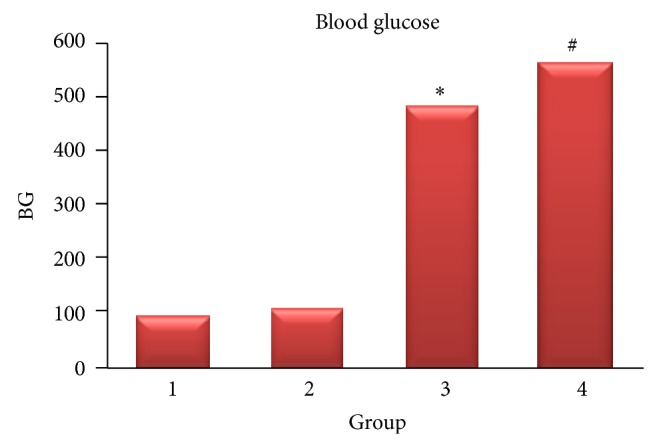
BG level. The blood glucose levels in vitamin C (3) and diabetic (4) groups were higher than in the control (1) and insulin (2) groups (*P* < 0.05)^∗#^.

At the end of 8 weeks, the BGs of vitamin C (487.5 ± 83.35 mg/dL) and diabetic (568 ± 45.2 mg/L) groups showed significant level of difference to the control (*P* < 0.001). The BG of insulin treated group (114.5 ± 15.3 mg/dL) showed no difference in comparison to the control group (101 ± 6.3 mg/dL) (*P* > 0.05) (Figure 1).

### 3.2. Plasma Cu Level

**Figure 2:**
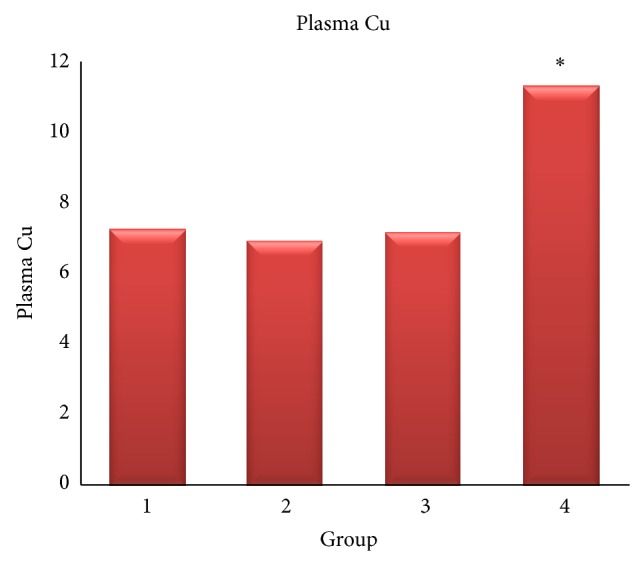
Plasma Cu level. Uncontrolled diabetic (4) group showed higher Cu level than other groups. Control (1), insulin (2), and vitamin C (3) ∗(*P* < 0.05).

The free plasma Cu level of control (7.29 ± 0.08 *μ*g/dL), insulin (7.29 ± 0.1 *μ*g/dL), and vitamin C (7.22 ± 0.11 *μ*g/dL) groups showed no significant level of difference (*P* > 0.05) (Figure 2).

### 3.3. Volume of Dentate Gyrus and CA3 Region

The volume of DG in diabetic (1.58 ± 0.04 mm^3^) and vitamin C (1.64 ± 0.04 mm^3^) groups showed significant level of difference compared with control (2.00 ± 0.09 mm^3^) and insulin (1.99 ± 0.07 mm^3^) groups (*P* < 0.001). The volume of CA3 in vitamin C (2.06 ± 0.12 mm^3^) and diabetic groups (1.98 ± 0.08 mm^3^) showed significant level of difference compared with control (3.15 ± 0.2 mm^3^) and insulin (3.08 ± 0.2 mm^3^) groups (*P* < 0.001). The volumes of DG and CA3 showed strong connection with BG level (*r* = −0.9). The connections between volumes of DG and CA3 with Cu were *r* = −0.6 and *r* = −0.7, respectively. Comparison between the total volume of DG and CA3 showed significant level of difference in control group (*P* < 0.001).

### 3.4. Numerical Density of Apoptotic Neurons (Figure 3)

The number of apoptotic neurons in DG of diabetic group (9615 ± 264 mm^−3^) showed significant level of difference (*P* < 0.001) in comparison with those of control (945 ± 106 mm^−3^), insulin (1074 ± 154 mm^−3^), and vitamin C (1119 ± 220 mm^−3^) groups. The numbers of apoptotic neurons in CA3 of diabetic group (18055 ± 3842 mm^−3^) showed significant level of difference (*P* < 0.001) in comparison to the control (613 ± 230 mm^−3^), insulin (616 ± 159 mm^−3^), and vitamin C (798 ± 137 mm^−3^) groups. The comparison between the number of apoptotic neuron in DG and CA3 was significant in control group (*P* < 0.05). The connection between the rate of apoptosis in DG and BG was *r* = 0.72 and for CA3 was *r* = 0.75. A strong connection was found between the rate of apoptosis in DG with plasma Cu level (*r* = 0.99) and in CA3 region (*r* = 0.99).

### 3.5. Response Index (RI)

**Figure 4:**
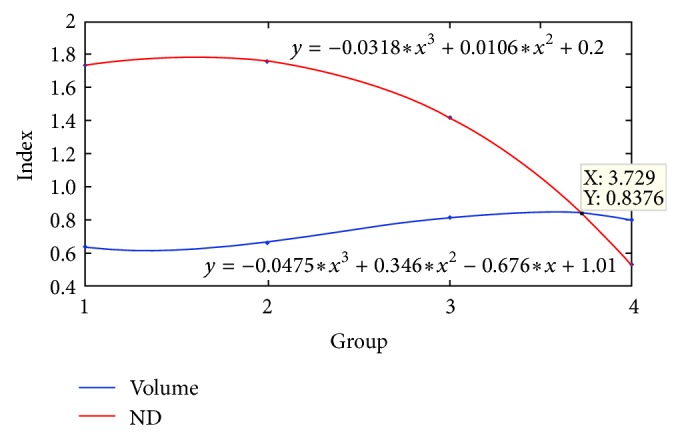
Equations of apoptosis numerical density (ND) and volume indices of groups. The tone of neuronal death shows more severe changes than those of volumetric changes. The point at which the two curves meet was calculated as *Y* = 0.83 (Con = 1, Ins = 2, VitC = 3 and Dia = 4). At this points devastating effects of diabetes mellitus reach an alarming rate.

RI of volumes (*V*_DG_/*V*_CA3_) of vitamin C and diabetic groups showed a clear difference in comparison with control (*P* < 0.05), while in contrast RI of apoptosis (ND_DG_/ND_CA3_) in diabetic group showed significant level of difference (*P* < 0.05) (Table 1). The equations of the two indices of interest are shown in Figure 4. Based on the equations, 0.08 is a critical point in which two curves met together.

## 4. Discussion

The results of this study showed that (1) DG and CA3 volume reduction begins and progresses independently of neuronal loss. (2) Volumetric index is more sensitive to diabetes mellitus than neuronal loss index. (3) Shifting of both indices toward 0.8 of RI is a critical reference point in which volumetric changes and neuronal loss become coincident. (4) Neuronal loss is more related to increased oxidative stress (plasma Cu level), while in contrast volume changes are more related to glycemic level. (5) Interestingly, 2 weeks of therapy with vitamin C maintained the Cu level and neuronal loss rate at a normal range but in contrast had no effect on BG and volume changes of the two subfields of interest. (6) Insulin therapy prevents degenerative changes of the dentate gyrus and CA3 regions and also maintains plasma Cu at normal level. (7) DM1 induces a higher neuronal loss in CA3 region in contrast to normal condition. For the first time, we suggested an index which has not been reported before. The index is based on functional and anatomic connectivity of two regions, DG to CA3. In fact, the index represents the degree of impression of two regions from each other in the presence of a disorder like diabetes mellitus. The index of the numerical density of neuronal loss indicates that (1) neuronal loss and volumetric changes at first are two separate scenarios. (2) The tone of neuronal loss in CA3 takes over that of DG, while the tone of volume changes in the DG and CA3 regions is more orchestrated in an insulin-dependent reductive manner. According to our results, the neuronal death could not be considered the main leading cause of volume change of the subfields of interest of hippocampus because by antioxidant therapy, vitamin C, in spite of the decreased rate of neuronal loss and normal plasma Cu level, the volumes of the two regions showed a clear reduction in comparison with insulin treated group. At present, we cannot explain the relation between the volume of the two regions of interest and exact mechanism(s) of exogenous insulin, but one of the possible mechanisms could be the dysregulation of hypothalamo-pituitary-adrenal (HPA) axis and subsequently an increase in circulating cortisol levels [12]. In part of our study, we weighed the adrenal glands of the animals and found that hyperglycemic animals had massive hypertrophy of the adrenal glands (unpublished data). So increased weight of adrenal gland (hypertrophy) could be considered as a mirror of HPA hyperactivity and lack of insulin [12]. According to the results of this study, neuronal loss and atrophy are two different but relevant scenarios. It seems that the lack of insulin imposed negative effects on granular cells of dentate gyrus and more vulnerability of pyramidal neurons of CA3 [18, 19]. Our results, in line with other studies, are indicative of neurotropic/protective role(s) of insulin on dentate gyrus and CA3 region and lack of insulin as a possible factor of neurodegeneration. Studies have reported that insulin replacement could reverse electrophysiological and morphological changes of hippocampus [20–22]. DG undergoes continuous proliferation through the life, and the rate of proliferation/death could be influenced by strenuous physical activity and hyperglycemia [12]. Hyperglycemia accelerates free radical generation and nonenzymatic degradation of ceruloplasmin which resulted in increased free plasma Cu level. Increased free Cu will interact with glycosylated proteins and cause more free radical generation [23]. The underlying repercussion of the increased free radical generation will result in more neuronal death through the complex mechanisms which can be considered as a hallmark of neurodegeneration [12]. At present, morphometric changes are detectable in clinical neuroimaging examinations, but neuronal loss even in large scale would not be discernible. Therefore, generalization of microscopic findings to neuroimaging findings can be worthwhile in evaluation of diabetic cases. The results of our study can be considered as a window to a comprehensive insight into the hippocampus encephalopathy. Although the generalization of this little study to human should be taken cautiously, more studies are needed in the future.

## Figures and Tables

**Figure 3:**
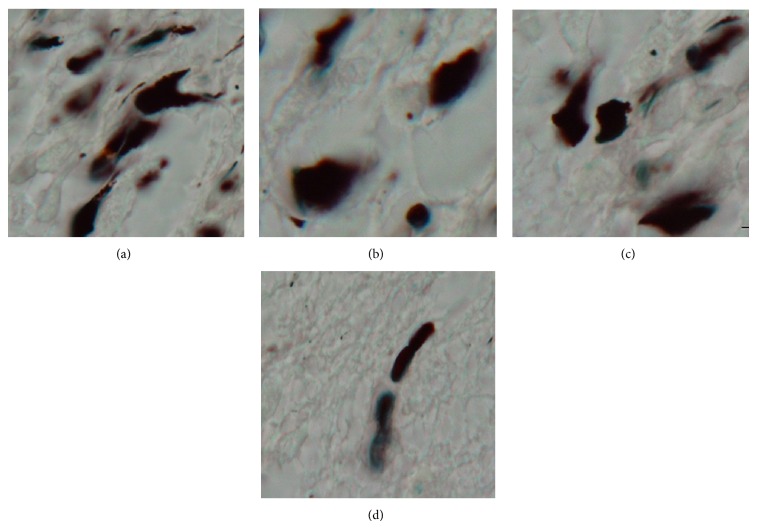
Apoptotic neurons stained darkly. Diabetic (untreated) (a), insulin group (b), vitamin C (c), and control groups (d) ×100.

**Table 1:** Response index of DG/CA3 for volume and numerical density of dark neurons. Volume index of diabetic and vitamin C (VitC) groups showed significant level of difference (^#∗^ *P* < 0.05). Numerical density index of diabetic (Dia) showed significant level of difference (^**^ *P* < 0.05).

Index	Con	Ins	VitC	Dia
*V*_DG_/*V*_CA3_	0.63 ± 0.00	0.64 ± 0.00	0.81 ± 0.01^*^	0.80 ± 0.00^#^
ND_DG_/ND_CA3_	1.54 ± 0.01	1.74 ± 0.02	1.4 ± 0.01	0.53 ± 0.0^**^
